# Traditional Chinese exercises on pain and disability in middle-aged and elderly patients with lumbar disc herniation: a systematic review and meta-analysis of randomized controlled trials

**DOI:** 10.3389/fmed.2023.1265040

**Published:** 2023-11-08

**Authors:** Weiye Zhang, Gewen Wang, Rong Xie, Jiawen Zhan, Liguo Zhu, Chunyou Wan, Hualong Xie, Chuhao Cai, Yuxuan Du

**Affiliations:** ^1^Third Department of Sports Medicine, Wangjing Hospital, China Academy of Chinese Medical Sciences, Beijing, China; ^2^Beijing Key Laboratory of Bone Setting Technology of Traditional Chinese Medicine, Beijing, China; ^3^Second Department of Spine, Wangjing Hospital, China Academy of Chinese Medical Sciences, Beijing, China; ^4^Department of Orthopedics, Tianjin Hospital, Tianjin, China

**Keywords:** lumbar disc herniation, Baduanjin, Yijinjing, Taichi, Wuqinxi, traditional Chinese exercises

## Abstract

**Background:**

Traditional Chinese exercises (TCEs) have played a significant role in treating various diseases. However, there is limited research assessing the efficacy of TCEs in treating Lumbar disc herniation (LDH). This study aimed to systematically evaluate the effects of four commonly used TCEs (Baduanjin, Yijinjing, Taichi, and Wuqinxi) on pain and disability in elderly patients with LDH.

**Objectives:**

To assess the quality of relevant randomized controlled trials (RCTs) to provide evidence support for the treatment of LDH.

**Methods:**

RCTs were identified through eight databases. Meta-analysis and trial sequence analysis (TSA) were conducted using RevMan 5.4, Stata 17.0, and TSA 0.9.

**Results:**

A total of 22 RCTs, involving 1931 patients, were included in the analysis. TCEs exhibited a superior effectiveness in treating LDH compared to the control group. However, the TSA analysis suggested the possibility of false positives, indicating the need for more high-quality RCT evidence. Nevertheless, TCEs showed reliable results in significantly improving the VAS score and JOA score of LDH patients.

**Conclusion:**

Current evidence indicates that the four TCEs have advantages in treating LDH in middle-aged and elderly individuals. However, considering the limitations of this study, we need to exercise caution in drawing conclusions, and further research is required to validate these findings.

**Systematic Review Registration:**

http://www.crd.york.ac.uk/PROSPERO, identifier [CRD42023431633].

## Graphical abstract

CNKI, China National Knowledge Infrastructure; Wan Fang, Wan Fang data; VIP, China Science and Technology Journal Database; CBM, Chinese Biomedical Literature Database; RCTs, randomized controlled trial; TSA, trial sequence analysis; VAS, visual analog scale; JOA, Japanese orthopedic association Scores; ODI, oswestry disability index; RR, response rate; SF-36, 36-Item Short Form Survey; MRMQ, Modified Roland-Morris Disability Questionnaire.

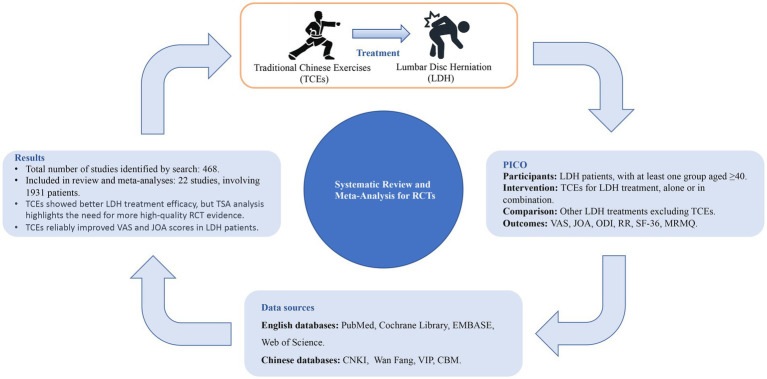

## 1. Introduction

Lumbar disc herniation (LDH) is a prevalent source of pain in the lower back and legs among adults. This condition significantly impacts their health and quality of life, placing a substantial burden on both society and families. LDH occurs when the nucleus pulposus of an intervertebral disc protrudes from its normal position, leading to the development of this ailment (1). Apart from lumbar spinal stenosis, spondylolisthesis, and fractures, herniated intervertebral discs have been found to be the primary cause of radicular pain (2).

Research has demonstrated that more than 90% of individuals within the middle-aged and elderly cohorts who suffer from LDH exhibit mechanical anomalies within their spinal and musculoskeletal structures (3). The lifetime prevalence of lumbar pain is 84%, with higher incidence rates among middle-aged and elderly populations, with 60% of patients also experiencing symptoms of leg pain (4). Chronic lumbar pain and limited lower limb function caused by LDH have led to poor quality of life and high disability rates among patients, indicating the need for treatment with high social costs (5).

However, China is also facing the enormous medical challenge brought about by population aging. According to survey and projected results, by 2050, there will be 400 million citizens in China aged 65 and above, including 150 million who are 80 years and older (6). Hence, as we aim to enhance the diagnosis and treatment of LDH, we must address its significant societal costs and explore cost-effective methods to ameliorate or cure symptoms in middle-aged and elderly LDH patients. LDH can be treated through surgical and non-surgical means, with most researchers advocating non-surgical options as the primary approach (7–8).

Traditional Chinese exercises (TCEs), through inheritance and reform, have been widely used as a conservative and complementary therapy in the treatment of mental disorders, endocrine disorders, orthopedic diseases, and the management of obesity (9–12). TCEs such as Baduanjin (BDJ), Yijinjing (YJJ), Taichi (TC), and Wuqinxi (WQX) have also been proven to improve pain and physical function in patients with LDH (13), showing good treatment effects in middle-aged and elderly patients (14, 15). TCEs present notable advantages. Firstly, they necessitate no specialized facilities or expensive equipment, allowing for flexibility in practice locations and timing. Secondly, TCEs effectively improve aerobic capacity, muscle strength, and balance. Thirdly, their low cost and straightforward technical requirements facilitate seamless community-wide adoption, endowing TCEs with considerable clinical value (16).

The evidence regarding the improvement of pain and disability in LDH through TCEs remains controversial. Therefore, the present systematic review aimed to assess the impacts of TCEs on pain and disability among middle-aged and elderly patients with LDH. This review offers evidence-based information that can be utilized in the clinical implementation of TCEs for LDH.

## 2. Methods

### 2.1. Data sources and search strategies

The Materials We conducted a comprehensive search across eight electronic databases, including four English databases (PubMed, Cochrane Library, EMBASE, and Web of Science) and four Chinese databases (China National Knowledge Infrastructure [CNKI], Wan Fang data [Wan Fang], China Science and Technology Journal Database [VIP], and Chinese Biomedical Literature Database [CBM]), from inception to May 1, 2023. No language restrictions were imposed. The search was performed by three researchers (WZ, GW, RX), and any discrepancies between the researchers were resolved through discussion. Additionally, all potentially relevant articles were examined and discussed to identify any additional studies.

The primary search terms used were (“traditional Chinese exercises” OR “qi gong” OR “Qigong” OR “Tai Chi” OR “tai ji” OR “ba duan jin” OR “Baduanjin” OR “yi jin jing” OR “Yijinjing” OR “Five-animal exercises” OR “wu qin xi” OR “Wuqinxi”) and (“lumbar disc herniation” OR “disc herniation”). To identify additional relevant studies, we examined the reference lists of relevant reviews. The World Health Organization International Clinical Trials Registry Platform (ICTRP) and the Chinese Clinical Trial Registry (ChiCTR) were also searched to identify ongoing or unpublished studies. In case of necessity, the reviewers made direct contact with the authors of the included studies. No restrictions were applied in terms of publication language or status.

### 2.2. Study selection and eligibility

#### 2.2.1. Types of studies

The objective of this meta-analysis was to examine the effectiveness of TCEs including BDJ, YJJ, TC, and WQX in reducing pain and disability levels in middle-aged and elderly patients with LDH. All the studies considered in this analysis were RCTs and were not restricted based on publication status. Excluded from the analysis were cross-sectional studies, animal experiments, systematic reviews and meta-analyses, disease guidelines, as well as studies for which we did not have access to the full texts.

#### 2.2.2. Types of participants

Participants included in the study had a clinical diagnosis of LDH, with at least one group having an average age of 40 years or older. There were no specific restrictions based on gender or nationality.

#### 2.2.3. Types of intervention

The control interventions encompassed various approaches such as waiting list, education, routine rehabilitation therapy, acupuncture, medicine, other modern exercise therapy, and alternative treatments that did not involve TCEs. It is crucial to highlight that TCEs were the exclusive exposure factor, with all other interventions remaining uniform across both groups.

#### 2.2.4. Types of measured outcomes

The focus of assessment will concentrate on evaluating the effectiveness of treatment through pre- and post-treatment efficacy evaluations, encompassing visual analog scale (VAS), Japanese orthopedic association Scores (JOA), and oswestry disability index (ODI). Additionally, secondary outcome measures of interest will comprise of response rate (RR), 36-Item Short Form Survey (SF-36), and Modified Roland-Morris Disability Questionnaire (MRMQ).

### 2.3. Data synthesis and extraction

The extraction of data was carried out independently by two reviewers (WZ, GW) according to pre-established criteria. The reviewers collected the following data: (1) fundamental details such as the first author’s name, publication year, and country; (2) participant characteristics including sample size and average age; (3) study design information such as interventions, outcomes, and duration of follow-up. Any disagreements were resolved through discussions between two reviewers and through communication with a third researcher (JZ). Duplicate articles were excluded using the EndnoteX20 software.

### 2.4. Literature quality assessment

Two researchers (WZ and GW) autonomously appraised the literature’s quality incorporated into this investigation, employing the methodological quality assessment criteria delineated in the Cochrane Handbook for Systematic Reviews of Interventions. A third researcher, JZ, was consulted to resolve any disagreements and finalize the results. The risk of bias assessments for all the included randomized controlled trials (RCTs) were reported as per the guidelines provided in the Cochrane Handbook for Systematic Reviews of Interventions. The criteria mainly considered were: (A) random sequence generation, (B) allocation concealment design treatment, (C) participant and rater blinding, (D) withdrawal and loss of follow-up, (E) incomplete outcome data and selective outcome reporting, and (F) other sources of bias. The evaluation methods employed were categorized as high risk of bias, low risk of bias, or unknown risk of bias.

### 2.5. Statistical analysis

The meta-analysis was performed using RevMan version 5.3 (The Cochrane Collaboration, Software Update, Oxford, United Kingdom). In the meta-analysis, the between-group mean differences of the studies for continuous outcomes were converted to the standardized mean difference (SMD) with 95% confidence intervals (CI). A random effects model was employed to account for clinical heterogeneity. In cases where there was no substantial heterogeneity (I^2^ ≤ 50%), a fixed-effects model was utilized. However, if significant heterogeneity was present, a random-effects model was applied. Subgroup analysis was conducted based on different types of control interventions and treatment effect modifiers. To assess the risk of publication bias, a funnel plot was used in meta-analyses including more than nine trials. Statistical significance was considered at *p* < 0.05. Trial Sequential Analysis (TSA) version 0.9 was used to perform trial sequence analysis.

## 3. Results

### 3.1. Study selection

**Figure 1:**
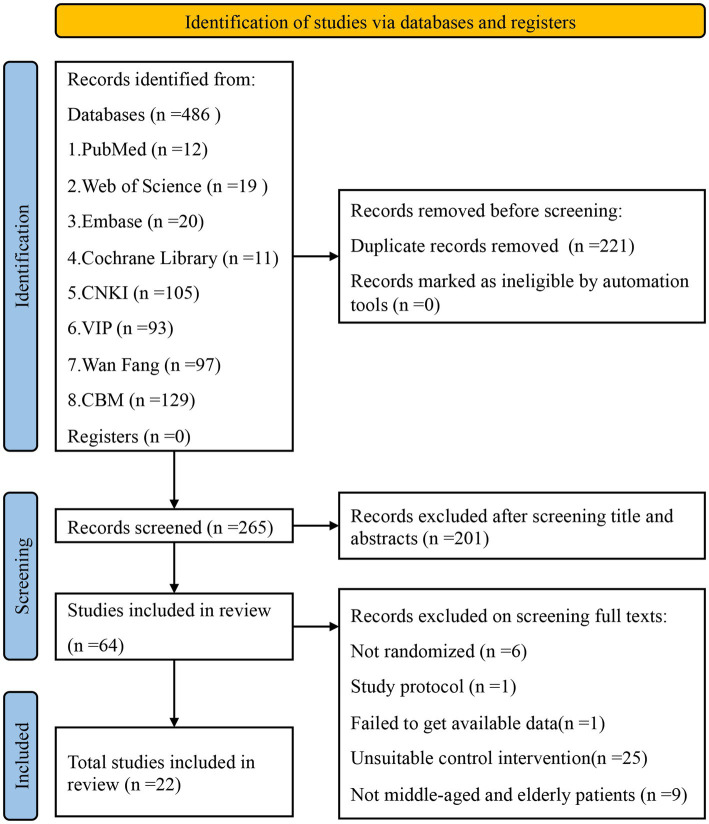
PRISMA flow chart of studies through the review. PRISMA, preferred reporting items for systematic reviews and meta-analyses.

At the beginning, 486 potentially relevant articles were gathered from the databases mentioned, namely PubMed, Cochrane Library, EMBASE, Web of Science, CNKI, Wang Fang, VIP, and CBM. Among them, 221 duplicate articles were eliminated by employing EndNote software for verification. By reviewing titles and/or abstracts, 201 articles that did not meet the criteria were excluded, resulting in 64 studies after the initial screening. After thoroughly examining the full text, 22 studies were ultimately included. Please review the PRISMA Flow Diagram (17) prepared in accordance with PRISMA guidelines (Figure 1).

### 3.2. Study characteristics

A total of 22 trials (18–39) with a parallel randomized control design were identified. In this review, a total of 1931 participants were included, with 964 in the experimental group and 967 in the control group. One study (22) was conducted in Singapore, while the remaining studies were conducted in China. Lastly, detailed characteristics of the 22 included trials were provided in Table 1.

**Table 1:** Summary table relating to study.

Study	Country	Year	Example		Average age (y)		Stochastic approach	Model of intervention		Ending indicator
			Exp	Con	Exp	Con		Exp	Con	
Yuan Guo et al.	China	2021	42	42	59.14 ± 4.62	59.90 ± 5.05	RNT	BDJ	Health education	ODI
Jiajun Kang et al.	China	2018	30	30	42.3 ± 6.1	45.4 ± 5.6	NA	BDJ + Conservative treatment	Backward walking exercise +Conservative treatment	VAS, JOA
Lei Li et al.	China	2022	48	48	58.46 ± 9.36	58.19 ± 9.12	RNT	BDJ	Health education	ODI, VAS, JOA, RR
Yubing Li et al.	China	2022	33	33	43.97 ± 4.35	44.28 ± 4.60	RNT	BDJ+ Chinese medicine	Five-point exercise + Chinese medicine	VAS, JOA, RR
Hairong Lin et al.	China	2018	30	30	40.44 ± 9.53	40.60 ± 7.21	NA	BDJ+ Control group	Massage therapy	VAS, RR
Xingxing Xu et al.	China	2018	45	45	45.27 ± 4.24	44.71 ± 4.42	RNT	BDJ+ Control group	Massage therapy	VAS, SF-36
Lei Liang et al.	China	2014	12	12	48 ± 6.12	48 ± 6.12	NA	BDJ	Massage therapy	RR
Hao Xu1 et al.	China	2015	30	30	48.33 ± 11.25	47.87 ± 10.20	NA	BDJ+ Control group	Conservative treatment	VAS, JOA
Qiangqiang Shang et al.	China	2014	30	30	42.20	42.20	NA	BDJ+ Control group	Massage therapy	VAS, JOA
Hao Xu2 et al.	China	2015	8	8	48.33 ± 11.25	47.87 ± 10.20	NA	BDJ+ Control group	Drug treatment	VAS, JOA
Longjiang Yang et al.	China	2018	40	40	43.5 ± 5.1	44.2 ± 5.3	RNT	BDJ+ Control group	Massage therapy	VAS, JOA, RR
Xiangbi Cai et al.	Singapore	2009	27	29	54.53 ± 11.25	52.46 ± 9.87	RNT	YJJ+ Control group	Traction therapy	VAS, MRMQ
Tao Ji et al.	China	2021	30	30	54.27 ± 5.46	54.87 ± 5.28	RNT	YJJ+ Control group	Massage therapy	VAS, JOA, RR
Yang Li et al.	China	2022	36	36	45.36 ± 11.38	46.75 ± 12.85	RNT	YJJ+ Control group	Massage therapy	SF-36, ODI
Zonghao Zhang et al.	China	2009	14	15	48.12 ± 14.37	49.53 ± 17.31	NA	YJJ+ Control group	Conservative treatment	VAS, JOA, RR
Yi Zhu et al.	China	2010	32	30	53.15 ± 13.62	52.83 ± 11.74	NA	YJJ	Traction therapy	VAS, MRMQ
Binghua Shao et al.	China	2022	30	30	40.83 ± 11.57	42.67 ± 11.37	medical record number	YJJ	Electric acupuncture	ODI, VAS, JOA, RR
Huijuan Si et al.	China	2022	40	40	41.49 ± 6.21	41.78 ± 6.19	RNT	YJJ+ Control group	Traction therapy	JOA, RR
Guangming Qing et al.	China	2012	77	80	43.5 ± 5.2	42.6 ± 4.7	NA	YJJ+ Control group	Regular treatment	VAS, JOA, RR
Qianyang Xia et al.	China	2014	150	150	47.3 ± 5.7	48.4 ± 5.3	NA	TC+ Control group	Massage therapy、acupuncture	JOA, RR
Xin Zhou et al.	China	2022	130	129	44.36 ± 10.44	51.77 ± 10.32	central randomization system	TC+ Control group	Massage therapy	ODI, VAS
Chunyan Ji et al.	China	2020	50	50	43.42 ± 3.11	43.45 ± 3.08	draw lots	WQX+ Control group	Regular treatment	RR

Exp, Experimental group; Con, Control group; RNT, random number table; VAS, visual analog scale; JOA, Japanese orthopedic association Scores; ODI, oswestry disability index; RR, response rate; SF-36, 36-Item Short Form Survey; MRMQ, Modified Roland-Morris Disability Questionnaire.

### 3.3. Quality assessment of the included literature

**Figure 2:**
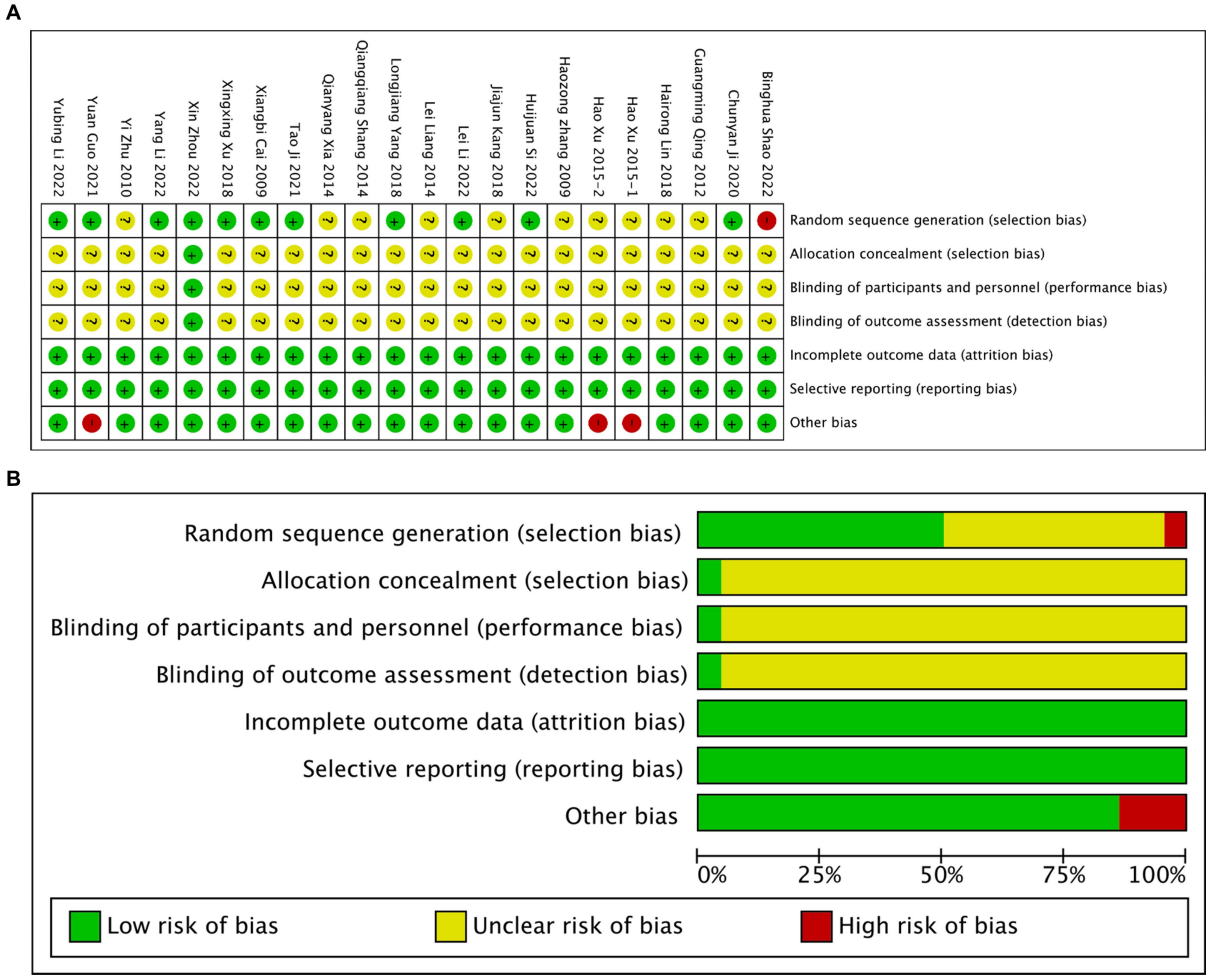
Risk of bias graph. **(A)** Bias risk for individual studies. **(B)** Bias risk for all studies.

The results of the literature quality assessment can be observed in Figure 2. All the trials included in this review were randomized, with nine of them employing random number tables for allocation (19–21, 23, 24, 26–28, 30–33). Only one trial (32) mentioned allocation concealment, which can be attributed to the unique nature of TCEs. It is important to note that blinding is challenging to implement in trials involving TCEs as an intervention. No relevant protocols were identified on the ClinicalTrials.gov website. In most studies, the intervention group received TCEs in combination with other treatments (such as traditional therapies, stretching, acupuncture, and medication therapy). These intervention groups were compared to control groups receiving corresponding traditional treatment methods. The frequency of treatment in the intervention group varied from three times per week to twice daily.

### 3.4. Effectiveness

#### 3.4.1. VAS score

**Figure 3:**
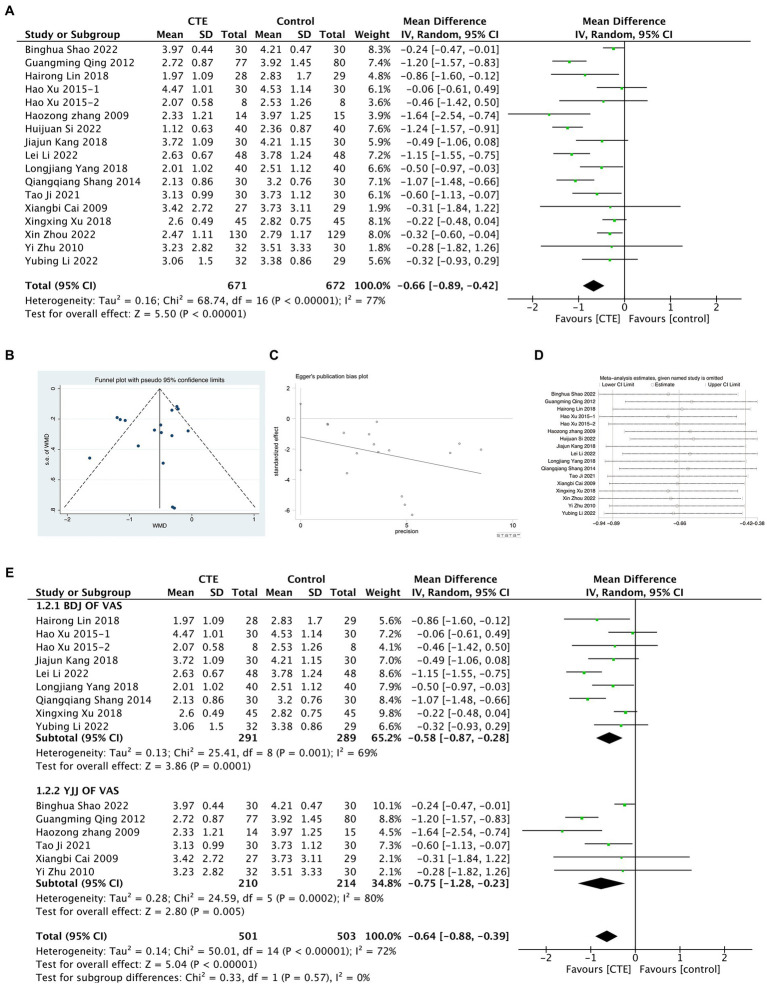
Meta-analysis results graph of VAS scores. **(A)** Forest plot comparing VAS scores between the treatment group and the control group. **(B,C)** Funnel plot of publication bias analysis results. **(D)** Sensitivity analysis results graph. **(E)** Forest plot of subgroup analysis between BDJ group and YJJ group.

In the studies included a total of 16 experiments utilized VAS score to evaluate the pain associated with LDH. The application of a random-effect model for meta-analysis revealed a statistically significant finding that the VAS score in the treatment group was lower than that of the control group [MD = −0.66, 95% CI (−0.89, −0.42) and *p* < 0.01; Figure 3A]. However, it is important to note that there was a high level of heterogeneity among the studies (*I*^2^ = 77%, *p* < 0.01). A funnel plot analysis demonstrated no evident publication bias (Begg’s Test *p* = 0.964, Egger’s test *p* = 0.253; Figures 3B,C). To further validate the results, a sensitivity analysis was conducted, and the outcomes indicated that although there was considerable heterogeneity among the studies, the results remained robust (Figure 3D). Based on the different types of TCES, a subgroup analysis was performed for exercise types, including 9 experiments focused on Baduanjin (BDJ) and 6 experiments on Yijinjing (YJJ), which were then included in the META analysis (Figure 3E). The results showed that the VAS scores of the BDJ group were significantly lower than those of the control group [MD = −0.58, 95% CI (−0.87, −0.28) and *p* < 0.01], and similarly, the VAS scores of the YJJ group were significantly lower than those of the control group [MD = −0.75, 95% CI (−1.28, −0.23) and *p* < 0.01]. A META regression analysis was conducted for the subgroups, and the results indicated [95% CI (−0.74, 0.40) and *p* = 0.536], signifying that there was no significant difference in the degree of improvement in VAS scores between the two groups.

#### 3.4.2. JOA score

**Figure 4:**
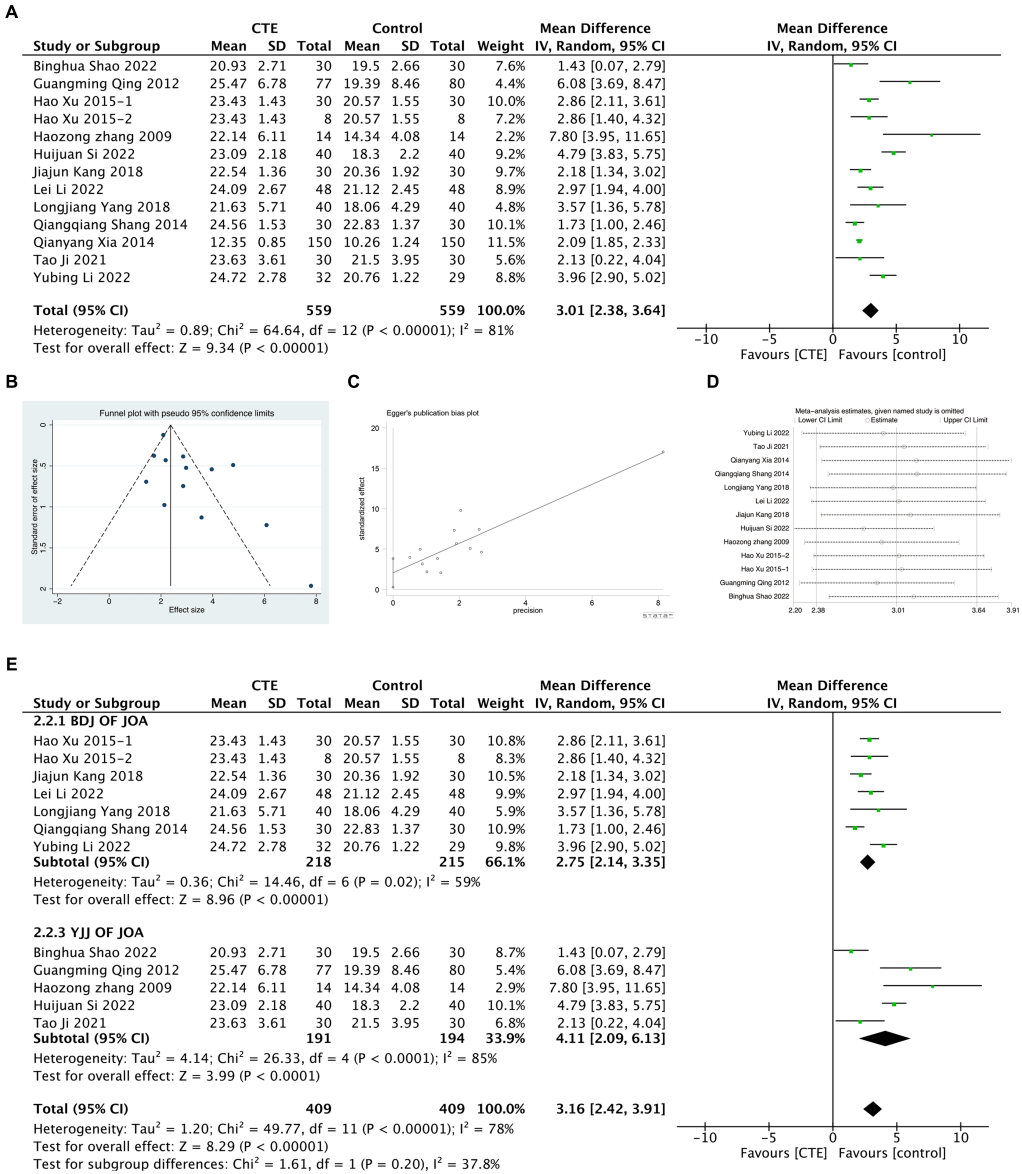
Meta-analysis results graph of JOA scores. **(A)** Forest plot comparing JOA scores between the treatment group and the control group. **(B,C)** Funnel plot of publication bias analysis results. **(D)** Sensitivity analysis results graph. **(E)** Forest plot of subgroup analysis between BDJ group and YJJ group.

A total of 13 experiments were included in the study to evaluate the functional impairment of LDH using the JOA score. Meta-analysis, employing the random effect model, indicated a significant difference in the JOA score between the treatment and control groups [MD = 3.01, 95% CI (2.38, 3.64), *p* < 0.01; Figure 4A]. However, considerable heterogeneity was observed among the studies (*I^2^* = 81%, *p* < 0.01). Publication bias was identified after conducting a funnel plot and tests of Begg and Egger (*p* = 0.127 and *p* = 0.026, respectively; Figures 4B,C). To address this bias, a sensitivity analysis was performed, confirming the robustness of the results despite the high heterogeneity (Figure 4D). Furthermore, subgroup analyses were carried out on studies with more than two experiments, based on different types of TCEs. The results demonstrated that the BDJ group exhibited a significantly higher JOA score than the control group [MD = 2.75, 95% CI (2.14, 3.35), *p* < 0.00001], whereas the YJJ group had a significantly lower JOA score [MD = 4.11, 95% CI (2.09, 6.13), *p* < 0.01]. Subgroup meta-regression analysis did not indicate a significant difference in the improvement of VAS between the two groups [95% CI (−0.97, 3.11), *p* < 0.01; Figure 4E].

#### 3.4.3. ODI score

**Figure 5:**
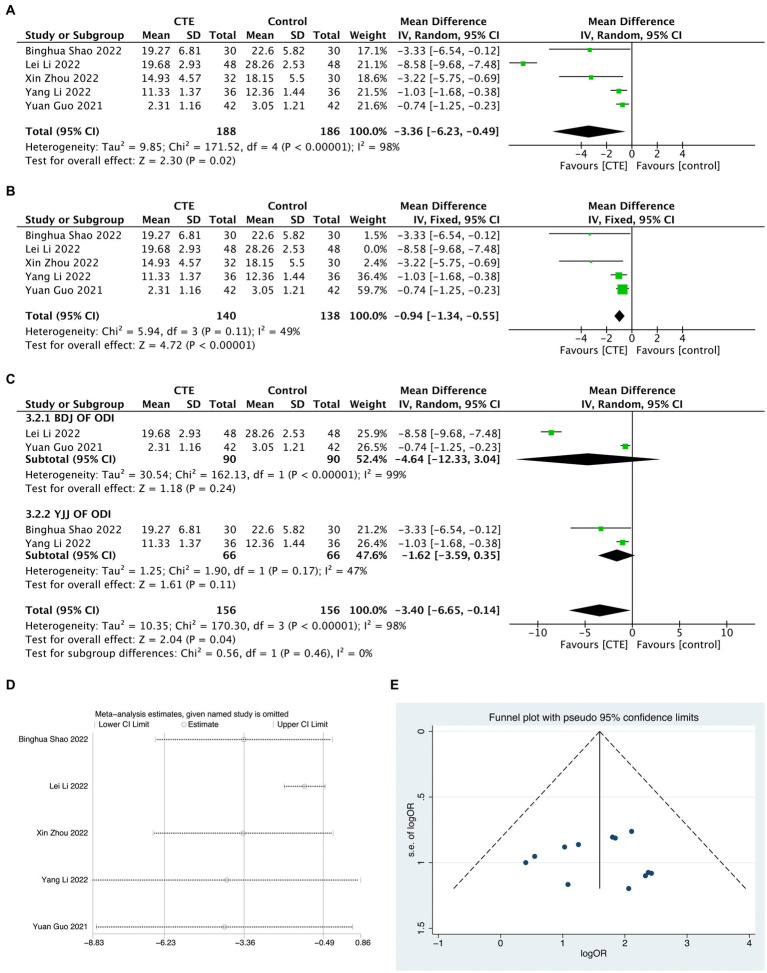
Meta-analysis results graph of ODI scores and response rate. **(A)** Meta-analysis results graph of ODI scores. **(B)** Forest plot of ODI scores after sensitivity analysis. **(C)** Forest plot of ODI scores after sensitivity analysis. **(D)** Sensitivity analysis results graph of ODI scores. **(E)** Funnel plot of publication bias analysis results for response rate.

A total of five experiments were included in the study, which utilized the ODI score. The meta-analysis, using a random-effects model, revealed a significant difference in the ODI score between the treatment and control groups, with statistical significance [MD = −3.36, 95% CI (−6.23, −0.49), *p* = 0.02, *I^2^* = 98%; Figure 5A]. Due to the limited number of included studies, the detection of publication bias was not conducted. However, the sensitivity analysis confirmed the robustness of the results (Figure 5D). Nevertheless, one study (23) demonstrated significant differences compared to the other four studies in the sensitivity analysis. Consequently, this study was excluded, and subsequent meta-analysis was performed, resulting in a significant decrease in heterogeneity [MD = −0.94, 95% CI (−1.34, −0.55), *p* < 0.01, *I^2^* = 49%; Figure 5B]. These findings indicated that the excluded study was the main source of heterogeneity. Additionally, subgroup analysis was conducted on studies that included two experiments, based on different types of TCEs. The analysis results revealed no significant differences in improving the ODI score compared to the control group for both the Baduanjin and Yijinjing exercise types. The combined subgroups exhibited high heterogeneity (*I^2^* = 98%), with the Baduanjin group demonstrating even higher heterogeneity (*I^2^* = 99%), further supporting the notion that the study conducted by Li (23) was the primary source of heterogeneity (Figure 5C). The meta-regression analysis for the subgroups showed no significant association between the heterogeneity among the studies and the type of exercise [95% CI (−15.54, 20.60), *p* = 0.608].

#### 3.4.4. Response rate

**Figure 6:**
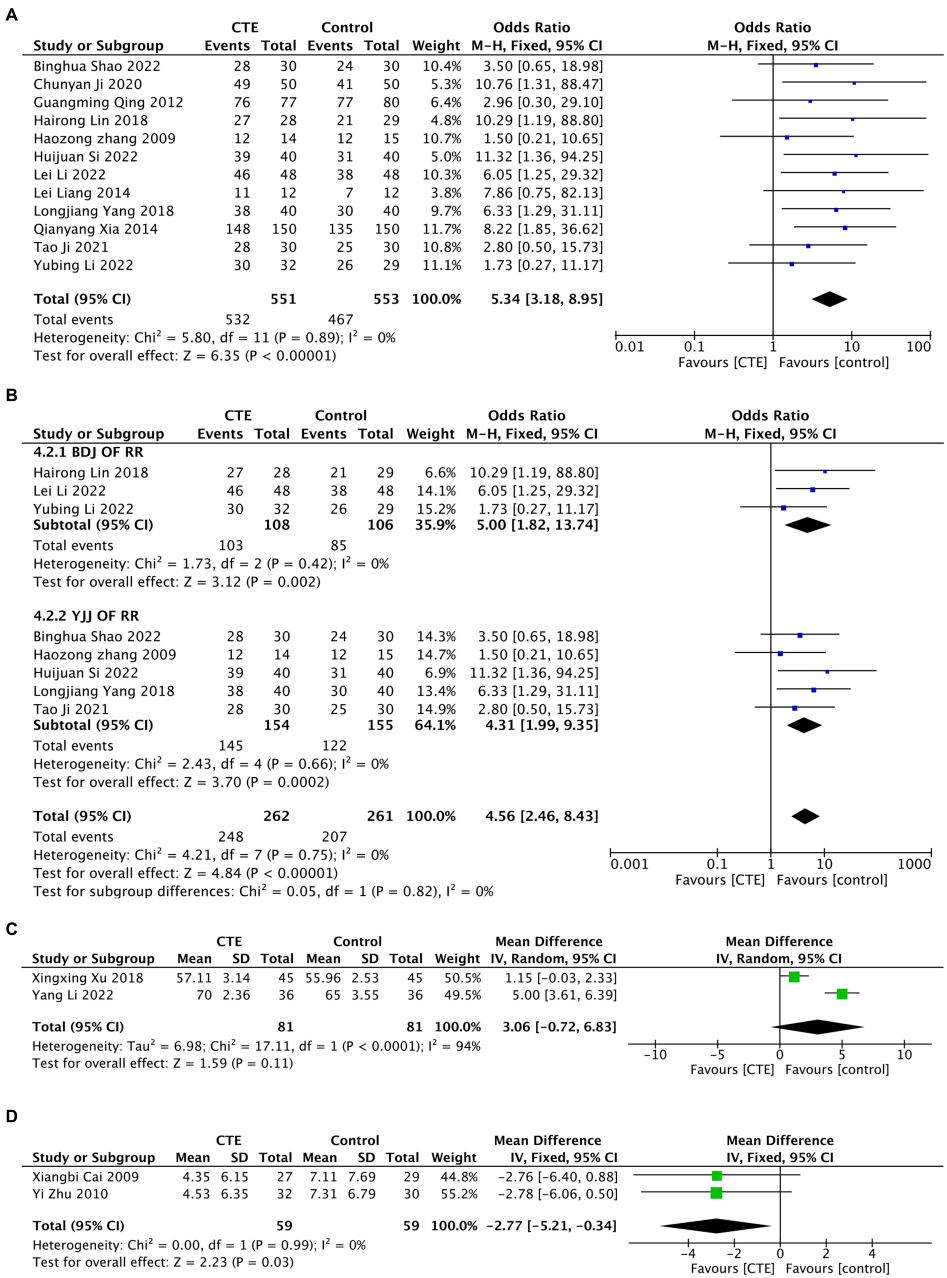
Meta-analysis results graph. **(A)** Forest plot of meta-analysis results graph for response rate. **(B)** Forest plot of subgroup analysis for response rate in BDJ group and YJJ group. **(C)** Forest plot of meta-analysis results graph for SF-36. **(D)** Forest plot of meta-analysis results graph for MRMQ scores.

A total of 12 studies were included in the analysis, using Response Rate to indicate treatment efficacy. Due to low heterogeneity after combining the studies (*I^2^* = 0%), a fixed-effect model was employed for the META analysis [OR = 5.34, 95% CI (3.18, 8.95), and *p* < 0.01; Figure 6A]. Funnel plot analysis did not reveal any significant publication bias (Begg *p* = 0.945, Egger *p* = 0.931; Figure 5E). Subgroup analysis was conducted for studies that included 2 experiments, and the results indicated that both Baduanjin and Yijinjing demonstrated significant effects on DLH (Figure 6B).

#### 3.4.5. SF-36 score

**Figure 7:**
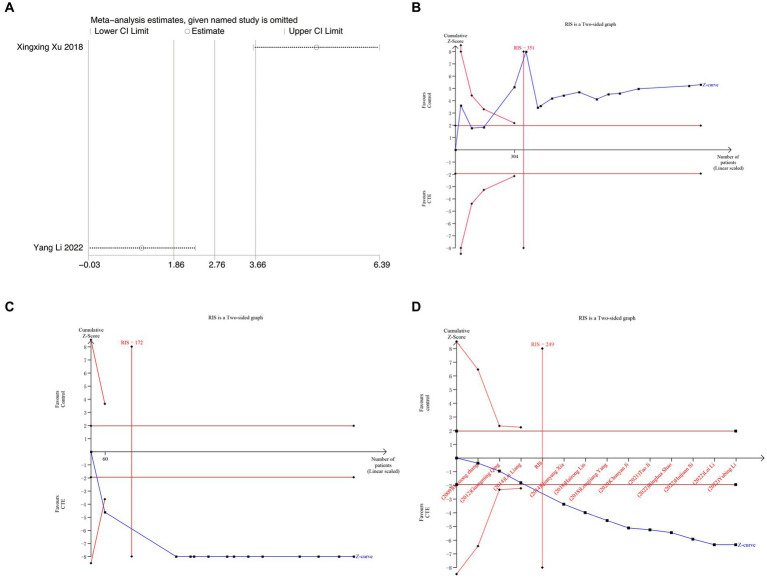
Meta-analysis and TSA analysis results graph. **(A)** Sensitivity analysis results graph for SF-36 scores. **(B)** VAS scores TSA analysis results graph. **(C)** TSA analysis results graph of JOA scores. **(D)** TSA analysis results graph of response rate.

Two studies using the SF-36 score were included in the analysis. Due to significant heterogeneity, a random-effects model was employed for the meta-analysis [MD = 3.06, 95% CI (−0.72, 6.83), and *p* = 0.11, *I^2^* = 94%; Figure 6C]. These findings suggest that TCEs may not effectively improve the SF-36 score for LDH. However, the sensitivity analysis indicated that the meta-analysis results were not robust, and the high heterogeneity may be attributed to the inclusion of two studies with different types of exercise and the limited number of studies included (Figure 7A).

#### 3.4.6. MRMQ score

Two studies were included in the analysis, and the MRMQ score was utilized in the research. Given the low heterogeneity, a fixed-effects model was employed for the meta-analysis, revealing a statistically significant reduction in SF-36 scores of LDH with TCEs [MD = −2.77, 95% CI (−5.21, −0.34), *p* = 0.03, *I^2^* = 0%; Figure 6D]. Furthermore, the patient function improved as a result.

#### 3.4.7. Safety

None of the 22 studies indicated that participants experienced any significant adverse reactions or uncomfortable symptoms.

### 3.5. Trial sequence analysis

Separate TSA was carried out for VAS, JOA, and RR. The required information size (RIS) was determined for each outcome measure: 351 for VAS, 172 for JOA, and 249 for RR. RIS was calculated by considering statistical indicators such as the type I error probability (α = 0.05), type II error probability (*β* = 0.2), mean difference, and variance data obtained from this meta-analysis. The results of TSA for VAS (Figure 7B) and JOA (Figure 7C) demonstrated that the cumulative Z value surpassed both the traditional and TSA thresholds, leading to a positive conclusion before the expected information quantity was reached. This suggests that CTEs exhibit a beneficial effect compared to the control group in reducing pain and improving function among LDH patients, and the evidence is conclusive. Nevertheless, the TSA results for RR (Figure 7D), which represents the efficacy rate, hinted at a possibility of false positive statistical difference. This indicates the necessity for future high-quality RCT studies to confirm the findings.

## 4. Discussion

### 4.1. Diagnosis of LDH

The diagnosis of LDH is challenging due to its multifaceted nature. The latest guidelines from the Japanese Orthopaedic Association emphasize a comprehensive approach, integrating symptoms, medical history, physical exams, and radiological findings. Consistency, particularly between physical exams and imaging, is crucial (40). The North American Spine Society similarly recommends a combined approach, using Manual Muscle Testing, Sensory Testing, Supine Straight Leg Raise, Lasegue Sign, and Crossed Lasegue Sign in conjunction with advanced imaging, primarily MRI, CT or CT myelography may complement when needed (41).

Prudent clinical judgment necessitates circumventing a sole reliance on radiological findings for diagnosis, as a comprehensive systematic study has illuminated the moderate diagnostic accuracy of the three imaging techniques (MRI, CT, CT myelography), potentially leading the clinician astray (42). Typical LDH symptoms include lumbar-sacral, lower limb, and perineal pain, sensory deficits, and, in severe cases, urinary and fecal dysfunction (43).

All patients included in this study had a confirmed diagnosis of LDH, albeit based on a comprehensive assessment of findings. Nevertheless, there exists inconsistency among the reference clinical practice guidelines consulted. Therefore, continuous updates to LDH clinical guidelines and intensified training of healthcare providers are imperative to enhance diagnostic consistency, ultimately fostering advancements in the treatment and research of LDH.

### 4.2. Clinical implications

The treatment of LDH is continually evolving, with two main approaches: conservative and surgical, as outlined in the latest clinical treatment guidelines (44). Conservative treatment remains the first-line therapy for LDH because symptoms tend to gradually improve as the herniated disc material causing them is absorbed. However, the choice between conservative and surgical treatments should be based on the patient’s individual circumstances and evidence from evidence-based medicine.

Lower back pain is one of the most common symptoms associated with LDH, with approximately 159.1 out of every 100,000 people worldwide seeking hospital treatment for disc-related lower back pain (45). It is typically categorized into no-radicular low back pain and radicular low back pain for differential treatment. Evidence-based medical research suggests that surgical treatment does not significantly outperform conservative treatment for no-radicular low back pain. However, for LDH accompanied by radicular low back pain, while conservative treatment can be effective (46), surgical treatment has short-term advantages in pain relief, although these advantages tend to diminish in long-term follow-ups (47, 48). For the elderly population under this study, evidence-based medicine research indicates that exercise can help alleviate the severity of lower back pain in older adults and may be considered as a medical intervention for this demographic (49).

However, it is important not to overlook the potential for exercise-induced biomechanical factors in the spine that can trigger or exacerbate lower back pain. Recent research by Baraldo et al. (50) on the relationship between lower back pain and strength training in elite athletes has found that the overall prevalence of lower back pain among athletes is at least 40%. In athletes engaged in high-intensity weightlifting, the probability of developing radiating lower back pain is as high as 54%. This may be attributed to the heavy loads placed on the spine during weightlifting, leading to increased risk of muscle injury and disc herniation, ultimately contributing to lower back pain. Their research also found that female athletes or older athletes have a higher incidence of illness. This suggests that doctors or physical therapists, when devising exercise plans for LDH patients, should consider both the nature of the exercise and the characteristics of the exercise population, to reduce the adverse effects of incorrect exercise.

Disability is one of the severe consequences of LDH, encompassing not only limited lumbar spine mobility, lower limb muscle weakness, and lower limb sensory impairment (40), but also a decrease in the patient’s ability to perform daily activities and interact socially due to LDH, leading to psychological disorders such as depression, insomnia, and reduced communication skills. This gradually evolves into a global public health issue awaiting better solutions, implying a significant societal cost (5). Multiple research findings suggest that when treating LDH and lower back pain, the patient’s psychological factors must be considered (51). Ballestra et al. (52) latest review found that for chronic lower back pain patients with a strong desire for pain relief, personalized counseling and treatment plans should be provided, adhering to a patient-centered care approach and a better understanding of the patient’s emotions and psychology, as patient expectations are significantly related to treatment outcomes. This suggests that physicians and family members, when combined with the correct treatment methods and strong patient motivation for recovery, can lead to a more optimistic prognosis.

TCEs are a form of patient-initiated physical activity that has gained widespread popularity in China and globally as a fitness regimen. Notable for their gentle exercise routines and moderate force application, TCEs have demonstrated applications and have been reported in the improvement of human balance, flexibility, skeletal muscle function, cardiovascular health, and respiratory system diseases, among others (53, 54).

### 4.3. TCEs treatment for LDH

TCEs, which encompass a wide range of exercise forms, were studied in this research, with a focus on the four most common forms: BDJ, YJJ, TC, and WXQ. Specifically, there were 11experiments on BDJ, 8 experiments on YJJ, 2 experiments on TC, and 1 experiment on WXQ. The mean age of the study participants was confined to individuals aged 40 years and older, as degenerative spinal changes leading to LDH are more prone to manifest in the middle-aged and elderly individuals. The analysis of the results showed promising findings, indicating significant therapeutic effects of all four TCEs in treating LDH. Notably, BDJ and YJJ demonstrated significant improvements in VAS scores and JOA scores of LDH. However, sub-group analysis revealed that these exercise modalities did not have a significant impact on ODI scores. This could potentially be attributed to the limited number of eligible studies and high heterogeneity, as determined through sensitivity analysis and META regression analysis. The statistical results for SF-36 and MRMQ scores varied, but caution is advised due to the limited number of studies included for these two indicators.

According to the existing literature, TCEs have the potential to alleviate pain and functional impairments in LDH patients through various mechanisms. Firstly, TCEs aid in the improvement of osteoporosis and the increase of bone mineral density. There is a widely recognized association between bone density and the occurrence of LDH (55, 56). As individuals age, a decline in bone mineral density becomes a major contributor to fractures and degenerative changes in the elderly. Therefore, enhancing or preserving bone density is a crucial treatment approach for LDH. For middle-aged and elderly individuals with low bone mass, exercise serves as an effective method to stimulate bone formation (57). TCEs, being aerobic exercises, have demonstrated the ability to regulate bone mineral density. For instance, BDJ applies pressure stimulation on the skeleton, which contributes to the regulation of bone mass (58). Regular and moderate TCEs can also modulate the endocrine system by promoting the secretion of hormones such as estrogen and irisin, which facilitate bone formation (59). As a result, this improvement in bone density can effectively alleviate patient pain (60).

Additionally, TCEs have been shown to improve muscle strength, enhance patient stability, and address mobility limitations. Moderate exercise with Tai Chi has been found to effectively improve balance and leg strength in patients (61). Furthermore, YJJ can improve skeletal muscle quality in middle-aged and elderly individuals with muscle loss (62). A study conducted in Spain revealed that Tai Chi significantly reduces acute pain in patients with muscle pain (63) and decreases the likelihood of falls and balance loss in older adults (64). Biomechanical experimentation showed that WQX, primarily performed while kneeling, can strengthen the lumbar muscles and increase spinal mobility (65). Moreover, a retrospective cohort study indicated that practicing Tai Chi can delay the imaging degeneration of LDH in middle-aged and older adults (15). As a complementary therapy, TCEs may also contribute to spinal balance (66) and provide relief from lower back pain (67). These studies collectively support the effectiveness of TCEs in improving pain and mobility limitations in patients.

In addition, there is evidence from evidence-based medicine supporting the benefits of TCEs in improving mental well-being (68), regulating the endocrine system (69), alleviating symptoms of arthritis (70), and helping middle-aged and elderly individuals reduce oxidative stress (71). For example, practicing BDJ can enhance cognitive function in older adults and alleviate musculoskeletal pain in chronic disease patients, thereby significantly improving sleep quality (72, 73). Enhancing mental well-being and regulating the endocrine system can significantly improve individuals’ daily life abilities, social interactions, and self-rehabilitation potential. Consequently, these improvements can provide valuable assistance in the rehabilitation of individuals with LDH.

This study has certain limitations. Firstly, among the RCTs included in this study, apart from one conducted in Singapore, the remaining 21 were all conducted in mainland China, which could introduce bias in the research results due to geographical constraints. Secondly, the number of studies for certain indicators is too limited to perform a more detailed and accurate data synthesis. The accuracy of the current stage’s data synthesis results is questionable, and further high-quality RCT research is needed in the future for validation. In the future, if more high-quality RCT articles are published, we will continue this study.

## 5. Conclusion

This study summarizes the evidence of TCEs treatment for Middle-Aged and Elderly Patients LDH from 22 RCT trials. Our preliminary meta-analysis suggests a significant efficacy of TCE in improving Pain and Disability, with no reported adverse events in the RCTs, indicating the preliminary safety of this treatment approach. However, caution must be exercised when interpreting these findings due to the limited number of studies for some outcome measures and the presence of high heterogeneity. In conclusion, the results of this study indicate a promising trend, suggesting that TCEs could be a feasible and relatively safe treatment option for LDH.

## Data availability statement

The original contributions presented in the study are included in the article/supplementary material, further inquiries can be directed to the corresponding authors.

## Author contributions

WZ: Data curation, Methodology, Writing – original draft. GW: Writing – original draft. RX: Writing – original draft. JZ: Funding acquisition, Writing – review & editing. LZ: Funding acquisition, Writing – review & editing. CW: Funding acquisition, Writing – review & editing. HX: Funding acquisition, Writing – review & editing. CC: Funding acquisition, Writing – review & editing. YD: Visualization and Writing – review & editing.
